# Molecular characterization of a novel subgenotype of lumpy skin disease virus strain isolated in Inner Mongolia of China

**DOI:** 10.1186/s12917-022-03383-5

**Published:** 2022-07-29

**Authors:** Xiaohui Zan, Haibi Huang, Yu Guo, Dongdong Di, Cun Fu, Shirong Wang, Youzhi Wu, Jialei Wang, Yan Wang, Yanhua Ma, Chunxia Chai, Rui Su, Qingqing Song, Wei Wang

**Affiliations:** 1 State Key Laboratory of Reproductive Regulation & breeding of grassland livestock, School of Life Sciences, Inner Mongolia University, Hohhot, China; 2 JINYU Biological Pharmaceutical Co., Ltd, Hohhot, China; 3 Inner Mongolia Autonomous Region Animal Disease Prevention and Control Center, Hohhot, China; 4 Basic Medical School, Inner Mongolia Medical University, Hohhot, China; 5 Beijing Boshi Biotech Co.Ltd, Beijing, China

**Keywords:** Lumpy skin disease virus, Molecular characterization, Recombination

## Abstract

**Background:**

The outbreak of Lumpy skin disease (LSD) in cattle caused by LSD virus (LSDV) was first reported in August 2019 in China. Since then, several LSD outbreaks have been reported in seven different provinces of China. Until now, several Lumpy skin disease virus (LSDV) strains from China have been reported and sequenced including LSDV/Xinjiang/2019 (MN598005.1), China/GD01/2020 (MW355944.1), and LSDV/Hongkong/2021 (MW732649.1). In October 2020, more than 1,700 cattle imported from Chile arrived in Xilingol, Inner Mongolia, and were diagnosed with LSD. Currently, limited data on the origin of the virus is available.

**Methods:**

Nucleotide sequences of the ORF11, ORF36, ORF74, ORF117, ORF126 genes and the complete genome of LSDV strains and isolates were downloaded from NCBI database. MEGA7.0 was used to perform phylogenetic analysis with Neighbor-Joining (NJ). DNASTAR software is used to analyze homologous comparison analysis with related genes of reference strains included in Genbank.

**Results:**

Compared with other strains isolated from China, the results of full genome sequence analysis showed the LSDV/NMG/2020 strain belonged to the recombinant strains. The LSDV/NMG/2020 strain is different from the current LSDV field isolates in Africa, the Middle East, Europe, and the newly emerged LSDV Russia variants. Based on the identities of P32, RPO30, EEV, GPCR and LSDV117 genes (99.8%, 99%, 99.8%, 99% and 98.7%), the sub-cluster recombinant containing LSDV/NMG/2020 strain is phylogenetically closer to the Russia strain (Saratov/2017).

**Conclusions:**

In this study, we reported a new isolated LSDV strain named LSDV/NMG/2020. The results of genomic characterization and phylogenetic analysis demonstrated that the LSDV/NMG/2020 isolate was a vaccine-like recombinant strain.

## Background

Lumpy skin disease (LSD) is a transboundary, viral disease of cattle with severe economic impact and is listed as a notifiable disease by the OIE[1]. LSD is caused by lumpy skin disease virus (LSDV), a pathogen of the genus Capripoxvirus of the family Poxviridae, the severity of clinical signs of LSD varies from subclinical to fatal depending on the virulence of the strains and the cattle breed’s susceptibility [2, 3]. Morbidity can range from 1% to almost 100%, with mortality most often between 1% and 3% [4]. It is mainly transmitted through insects and does not infect humans, but could spread to cattle, water buffalo and certain antelopes[5]. LSD was first discovered in Zambia in 1929. In 1988, the first report in Egypt from where it spread in 1989 to the Middle East. Intercontinental transmission occurred again in 2015 [6, 7], and now it was expanded into Greece, eastern Europe, Russia and India [8–10]. But lumpy skin disease virus was eradicated in Greece and eastern Europe using vaccination. LSD was reported in China and neighbouring countries of Indian and Vietnam during 2019–2020 [11, 12].

A live-attenuated vaccine, based on the Neethling-type field strain was developed by multiple passages in lamb kidney cells and chorioallantoic membranes of embryonated eggs in the 1950s in South Africa [13]. Live attenuated LSD vaccines with the strain of the “Neethling” as a prototype has been used in Africa for over 50 years and are now widely used in most affected countries [14, 15]. In 2017, a vaccine-like strain has been discovered linked to circulating to LSD outbreaks in Russia [16]. A novel LSDV strain, known as Saratov/2017, from diseased cattle in southern Russia in 2017 revealed similarities with the Neethling and KSGPO-like vaccine strains [17]. This is the first report demonstrating that an attenuated LSD vaccine strain has been identified in Russian cattle given the ban on the use of live attenuated vaccines against LSDV[18]. After the introduction of vaccines in Kazakhstan in 2017, vaccine-like isolates underwent multiple dispersal events across a wide area toward the East along the Russian border, resulting in a new epidemiological wave of genetically different LSDV[8, 19, 20].

In 2019, LSD was introduced to the Xinjiang region of China, which then swept through a vast geographic area in China [21]. In 2020, seven provinces and cities in Xinjiang, Fujian, Jiangxi, Guangdong, Anhui, Zhejiang and Taiwan have all experienced epidemics [11]. In October 2020, more than 1,700 cattle imported from Chile arrived in Xilingol, Inner Mongolia, and were diagnosed with LSD. At the same time, cattle destined for the Baotou city, Ningxia province and Shandong province were also affected. Currently, limited data on the origin of the virus is available. Here, we reported phylogenetic analysis of the genome of LSDV LSDV/NMG/2020 strain reported by China. LSDV/NMG/2020(ON616408), LSDV/Xinjiang/2019(MN598005.1), China/GD01/2020 (MW732649.1), 20L43Ly-Quoc/VITM/20(MZ577074.1), and LSDV/Hongkong/2020 (MW732649.1) belong to the same branch. These strains are different from all previously reported LSDV strains, but the cluster is a new lineage.

## Materials and methods

### Clinical histories and collections of samples

**Fig. 1:**
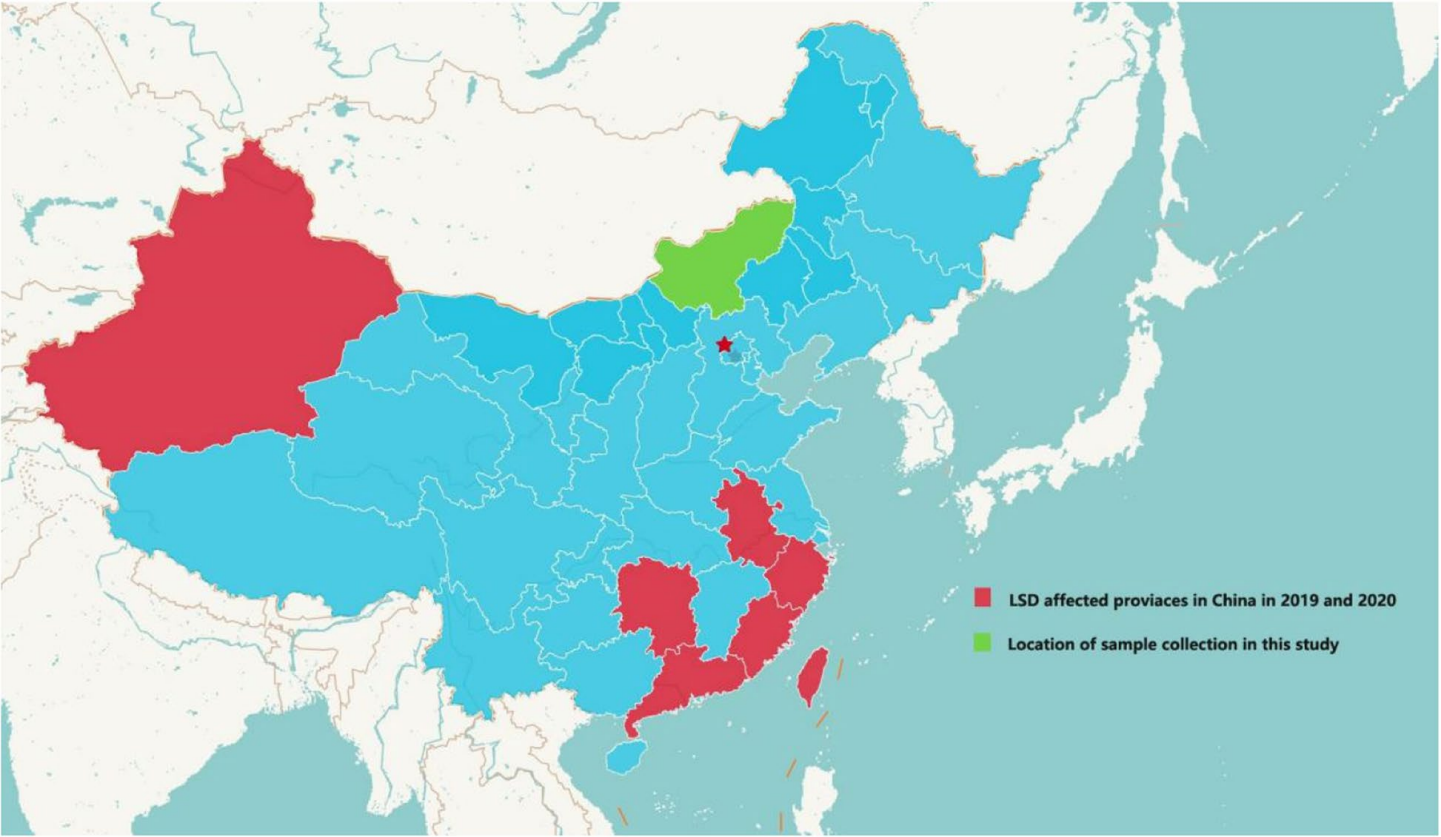
Location of study area. Districts where outbreaks occurred are shown in green range (Xilingol, Inner Mongolia)

Samples of cattle (*n* = 3) collected in Xilingol City (Fig. 1) were collected aseptically in a sterile tube containing a virus transport medium and stored at 4 °C. These samples were then transported to the third-level biological safety protection laboratory (BSL3) of Jinyu Paulin Biopharmaceutical Co., Ltd on ice.

### Sample processing

The samples were processed by cutting up the scab tissue with a sterile scalpel and tweezers, pulverizing with sterile sand using a sterile mortar and pestle, and preparing a suspension in DMEM containing antibiotics. The tissue homogenate was submitted to two freeze-thaw cycles to release the intracellular virus followed by centrifugation at 5,000 rpm for 10 min at 4 °C to remove any coarse particles. Collect the supernatant of the tissue homogenate in a separate sterile tube for detection of LSDV by PCR and virus isolation. The sample processing process is carried out in the third -level biological safety protection laboratory (BSL3) of Jinyu Paulin Biopharmaceutical Co., Ltd.

### Agents identification

Total DNA was extracted from 600 µl of clinical specimens using a QIAamp DNA Mini Kit following the manufacturer’s instructions. Extracted DNA was quantified using NanoDrop One. This was subjected to the P32 gene targeting PCR to identify and discriminate LSDV using the primers and protocol designed by OIE: F-5’-TTT CCT GAT TTT TCT TAC TAT-3’; R-5’-AAA TTA TAT ACG TAAATAAC-3’. Amplification was performed using the following conditions: initial denaturation cycle at 95℃ for 5 min, 34 cycles (denaturation at 95℃ for 45 s, annealing at 50℃ for 30 s, and extension at 72 ℃ for 1 min), followed by a final extension cycle at 72℃ for 5 min. The PCR assay was performed in 25 µL volume, including 12.5 µL Dream Taq green PCR master mix (2×) (Thermo Scientific, Germany), 1 µL of each primer (10 pmol/µl), 8.5 µL deionized water, and 2 µL of DNA template.

### Nucleotide sequencing

In order to further confirm the identity of the virus of LSDV/NMG/2020, duplicate samples were submitted for complete genome sequencing and only high-quality sequences were submitted to Genbank database with Accession Numbers of DN616408 (Lumpy skin disease virus strain LSDV/NMG/2020, complete genome), OL977078 (GPCR), OL977075 (EEV), OL977074 (P32), OL977077 (LSDV117) and OL977076 (RPO30) .

### Phylogenetic analysis

Nucleotide sequences of the ORF11, ORF36, ORF74, ORF117, ORF126 genes and the full-length genes of LSDV strains and isolates were downloaded from NCBI database. Multiple alignments of these sequences were performed by MEGA 7.0 with ClustalW method. MEGA7.0 was used to perform phylogenetic analysis with Neighbor-Joining (NJ). The bootstrap values were determined from 1000 replicates of the original data. DNASTAR software is used to analyze homologous comparison analysis with related genes of reference strains included in Genbank.

## Results and discussion

The earliest recorded LSD outbreak occurred in several epidemics in seven provinces were confirmed and caused considerable economic losses to the cattle industry in China. Currently, the sequences of three strains, LSDV/Xinjiang/2020 (MN598006.1), China/GD01/2020 (MW355944.1), LSDV/Hongkong/2020 (MW732649.1) have been reported in China. This study investigated the molecular characterization of the new isolated LSDV/NMG/2020 strain has been analyzed and compared with vaccine and wild-type strains of China, Russia, Indian, Vietnam, and several other countries.

**Fig. 2:**
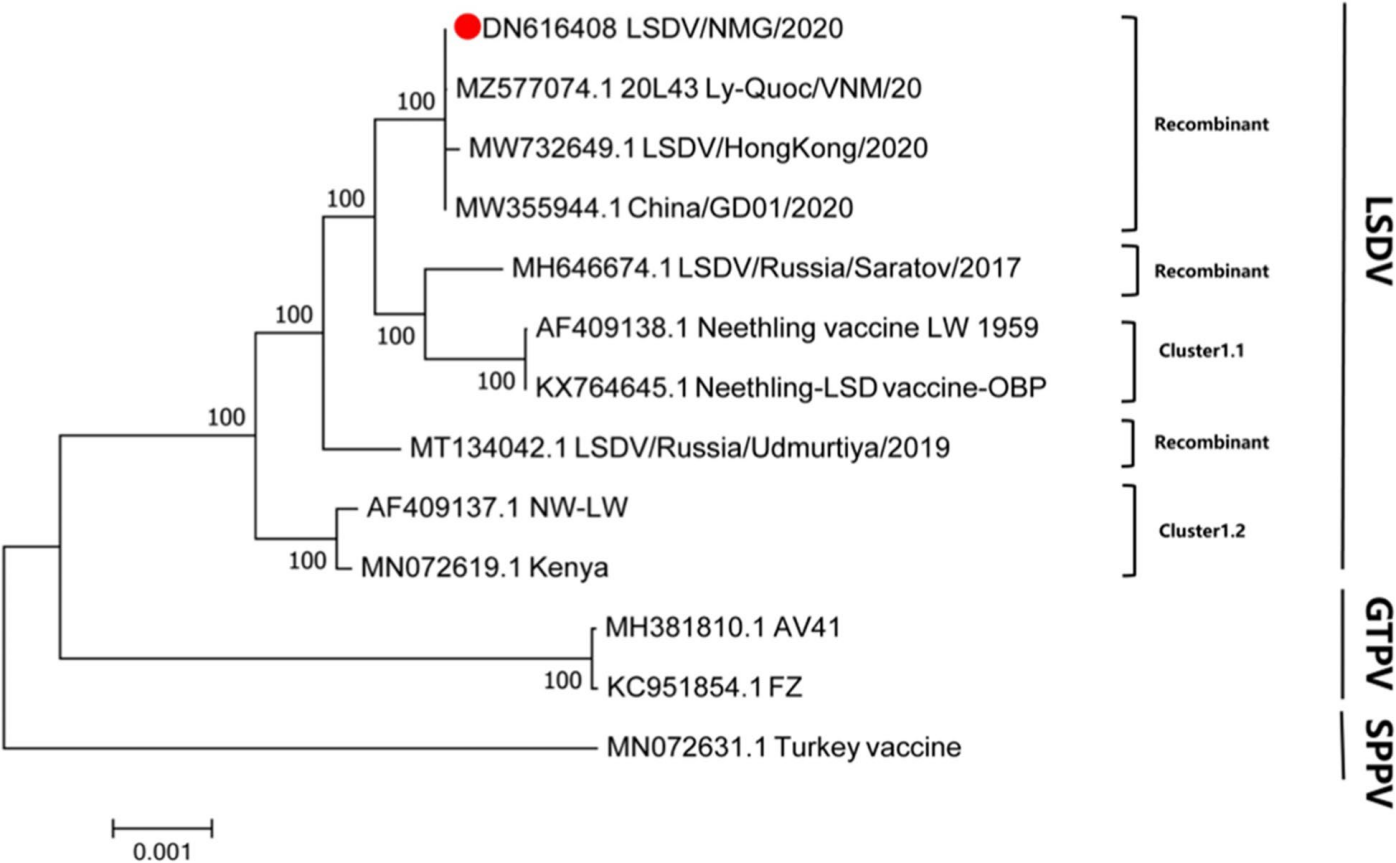
Phylogenetic tree with Neighbor-Joining (NJ) shows the relationship between LSDV full-length genomic sequences from LSDV/NMG/2020, marked with red round, with other Capripoxvirus gene sequences from GenBank

**Fig. 3:**
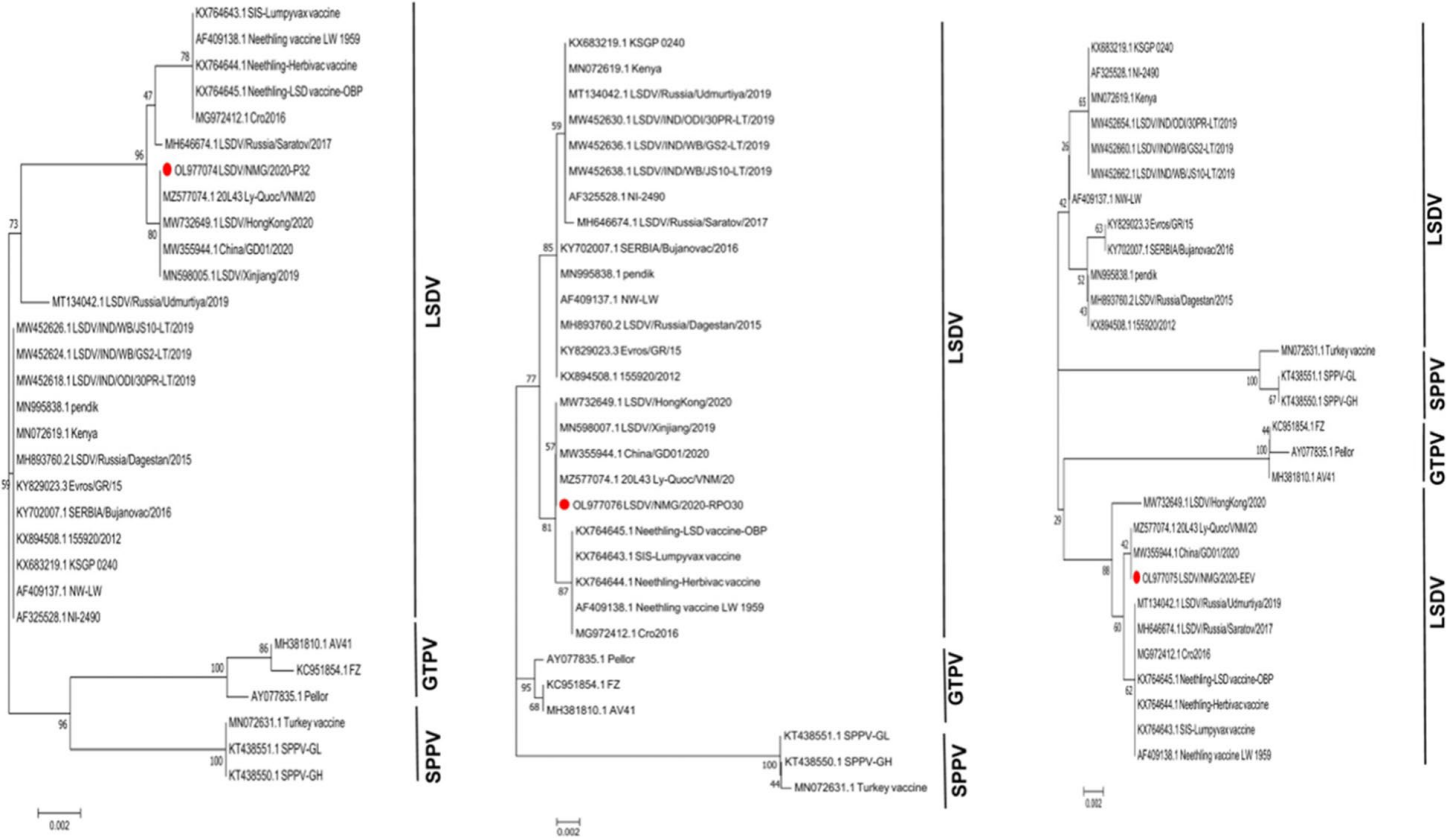
Phylogenetic trees with Neighbor-Joining (NJ) show the relationship between LSDV P32, RPO30 and EEV genomic sequences from LSDV/NMG/2020, marked with red round, with other Capripoxvirus gene sequences from GenBank

**Fig. 4:**
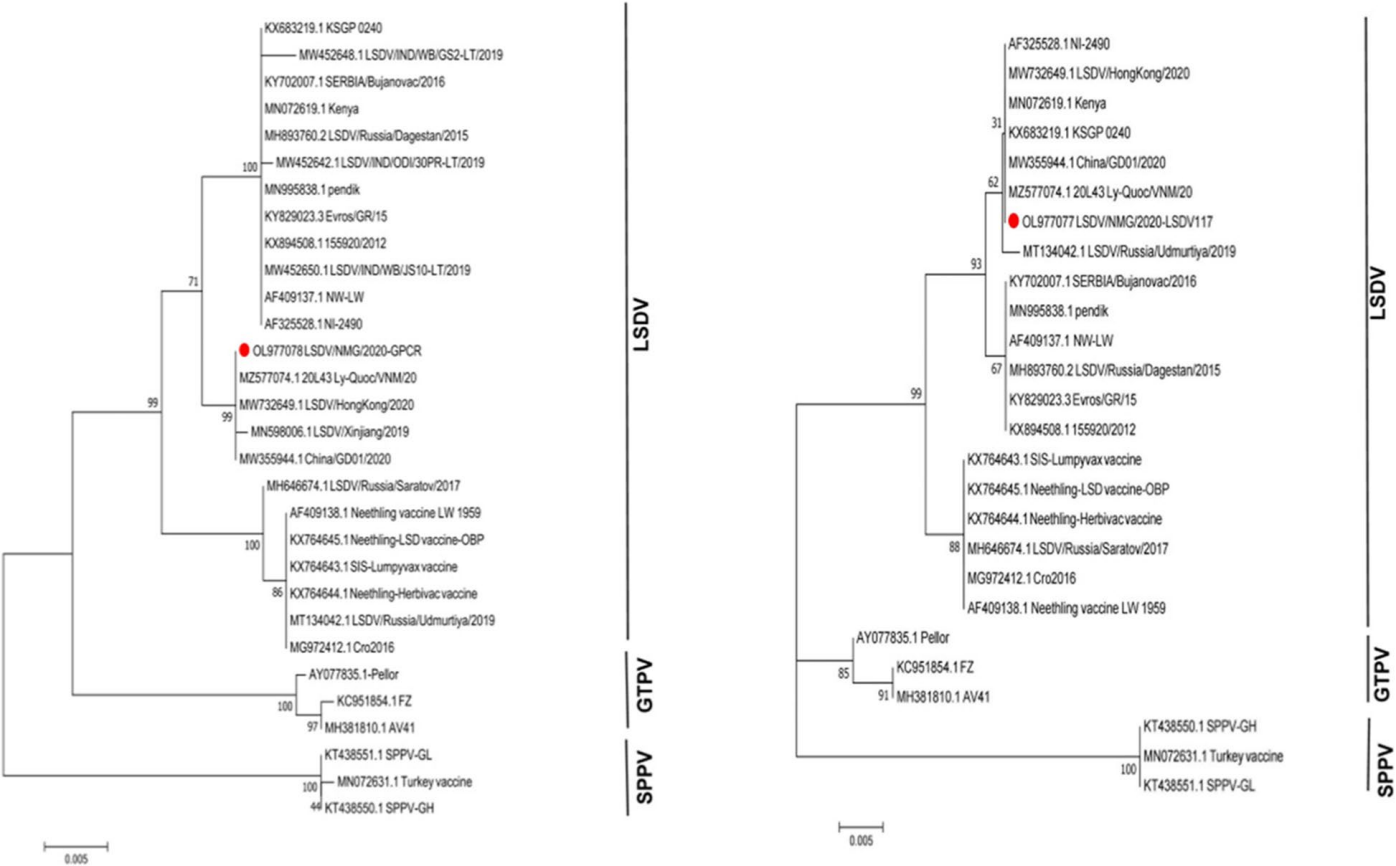
Phylogenetic trees with Neighbor-Joining (NJ) show the relationship between LSDV GPCR and LSDV117 genomic sequences from LSDV/NMG/2020, marked with red round, with other Capripoxvirus gene sequences from GenBank

The previous studies have demonstrated that the LSDV virus strains could be divided into two major subgroups[22]. In this study, the full-length genomic analysis showed that all strains isolated from China have been clustered into the same lineages (Fig. 2). Significantly, these strains in the same lineages were assembled into a new cluster, which is different from the vaccine and the current wild-type strains. According to the full-length sequence, LSDV/NMG/2020 belongs to the same sub-cluster as 20L43Ly-Quoc/VNM/20(MZ577074.1), China/GD01/2020(MW732649.1) and LSDV/HongKong/2020 (MW732649.1). Although they both belong to recombinant strains, LSDV/NMG/2020, LSDV/Russia/Udmurtiya/2019 and LSDV/Russia/Saratov/2017 belong to different branches. It is worth noting that the sequences based GPCR, LSDV117 is highly similarity with 99%, 99.6% with field-type, and less similar with 98.9%, 98.7% compared with the Neethling/LW-1959/vaccine strains. Base on the RPO30, P32, and EEV genes, it was more similar with 99.7%, 99.8%, and 99.8% compared with the Neethling/LW-1959/vaccine and less similar with 99.3%, 99.3%, 94.1% compared to wildtype strains (Table 1). Interestingly, the LSDV/NMG/2020 strain has the characteristics of the Neethling/LW-1959/vaccine and KSGPO-like strains in the virus genome. It was identified as having the P32, RPO30 and EEV genes derived from the vaccine strains and the genes of GPCR and LSDV117 derived from field isolates (Figs. 3 and 4). The phenomenon of recombination within the family Poxviridae has been reported [23]. Two novel recombinant LSDV strains generated from NI-2490/KSGPO-like and Neethling/LW-1959/vaccine isolates named Saratov/2017 and Russia/Udmurtiya/2019 were isolated in Russia and clustered into the different branches according to the virus genes. The LSDV RUSSIA/Saratov/2017 strain is more closely genetically related to the Neethling/LW-1959/vaccines compared (> 99.3%), than the field strains (> 98.64%) [17]. The 2019-2020 outbreak of the LSDV strain in China was not identical to the outbreak in India (Table 1). The origin of the LSDV in these outbreaks is still unclear.

**Table 1:** The nucleotide homology between LSDV/NMG/2020 and other Capripoxvirus gene sequences

	Genbank ID	Abbreviation name	virus type	Year	Origin	Wildtype or vaccine	Identity to Inner Mongolia				
							GPCR	RPO30	P32	EEV	LSDV117
1	KX764645.1	Neethling-LSD_vaccine-OBP	LSDV	U	South Africa	vaccine	98.9	99.7	99.8	99.8	98.7
2	KX764644.1	Neethling-Herbivac_vaccine	LSDV	U	South Africa	vaccine	98.9	99.7	99.8	99.8	98.7
3	KX764643.1	SIS-Lumpyvax_vaccine	LSDV	U	South Africa	vaccine	98.9	99.7	99.8	99.8	98.7
4	AF409138.1	Neethling_vaccine_LW_1959	LSDV	U	South Africa	vaccine	98.9	99.7	99.8	99.8	98.7
5	MG972412.1	Cro2016	LSDV	U	Croatia	vaccine	98.9	99.7	99.8	99.8	98.7
6	MN072619.1	Kenya	LSDV	1958	Kenya	Wild type	99.1	99.2	99.3	94.1	100
7	AF325528.1	NI-2490	LSDV	1958	Kenya	Wild type	99.1	99.2	99.3	94.1	100
8	KX683219.1	KSGP_0240	LSDV	1974	Kenya	vaccine	99.1	99.2	99.3	94.1	100
9	AF409137.1	NW-LW	LSDV	1999	South Africa	Wild type	98.1	99.3	99.3	94.3	99.6
10	KX894508.1	155,920/2012	LSDV	2012	Israel	Wild type	98.1	99.3	99.3	94.1	99.6
11	MN995838.1	pendik	LSDV	2014	Turkey	Wild type	98.1	99.3	99.3	94.1	99.6
12	MH893760.2	LSDV/Russia/Dagestan/2015	LSDV	2015	Russia	Wild type	98.1	99.3	99.3	94.1	99.6
13	KY829023.3	Evros/GR/15	LSDV	2015	Greece	Wild type	98.1	99.3	99.3	94	99.6
14	KY702007.1	SERBIA/Bujanovac/2016	LSDV	2016	Serbia	Wild type	98.1	99.3	99.3	94	99.6
15	MH646674.1	LSDV/Russia/Saratov/2017	LSDV	2017	Russia	Wild type	99	99	99.8	99.8	98.7
16	MT134042.1	LSDV/Russia/Udmurtiya/2019	LSDV	2019	Russia: Udmurtiya	Wild type	98.9	99.2	99.1	99.8	99.8
17	MW452642	LSDV/IND/ODI/30PR-LT/2019	LSDV	2019	India	Wild type	99	/	/	/	/
18	MW452630	LSDV/IND/ODI/30PR-LT/2019	LSDV	2019	India	Wild type	/	99.2	/	/	/
19	MW452618	LSDV/IND/ODI/30PR-LT/2019	LSDV	2019	India	Wild type	/	/	99.3	/	/
20	MW452654	LSDV/IND/ODI/30PR-LT/2019	LSDV	2019	India	Wild type	/	/	/	94.1	/
21	MW452648	LSDV/IND/WB/GS2-LT/2019	LSDV	2019	India	Wild type	98.9	/	/	/	/
22	MW452636	LSDV/IND/WB/GS2-LT/2019	LSDV	2019	India	Wild type	/	99.2	/	/	/
23	MW452624	LSDV/IND/WB/GS2-LT/2019	LSDV	2019	India	Wild type	/	/	99.3	/	/
24	MW452660	LSDV/IND/WB/GS2-LT/2019	LSDV	2019	India	Wild type	/	/	/	94.1	/
25	MW452650	LSDV/IND/WB/JS10-LT/2019	LSDV	2019	India	Wild type	99.1	/	/	/	/
26	MW452638	LSDV/IND/WB/JS10-LT/2019	LSDV	2019	India	Wild type	/	99.2	/	/	/
27	MW452626	LSDV/IND/WB/JS10-LT/2019	LSDV	2019	India	Wild type	/	/	99.3	/	/
28	MN452662	LSDV/IND/WB/JS10-LT/2019	LSDV	2019	India	Wild type	/	/	/	94.1	/
29	MN598006.1	LSDV/Xinjiang/2019	LSDV	2019	China	Wild type	99.9	/	/	/	/
30	MN598007.1	LSDV/Xinjiang/2019	LSDV	2019	China	Wild type	/	100	/	/	/
31	MN598005.1	LSDV/Xinjiang/2019	LSDV	2019	China	Wild type	/	/	100	/	/
32	MW355944.1	China/GD01/2020	LSDV	2020	China	Wild type	100	100	100	100	100
33	MW732649.1	LSDV/HongKong/2020	LSDV	2020	China	Wild type	100	100	100	99.6	100
34	MZ577074.1	20L43_Ly-Quoc/VNM/20	LSDV	2020	VietNam	Wild type	100	100	100	100	100
35	MN072631.1	SPPV-Turkey vaccine	SPPV	U	Turkey	vaccine	92.5	94.7	98	97.1	94.2
36	KT438551.1	SPPV-GL	SPPV	2013	China	Wild type	93.4	94.9	98	97.1	94.2
37	KT438550.1	SPPV-GH	SPPV	2013	China	Wild type	93.4	94.9	98	97.1	94.2
38	AY077835.1	GTPV-Pellor	GTPV	2000	Kazakhstan	Wild type	96.3	98.5	98.3	97.1	97.1
39	KC951854.1	GTPV-FZ	GTPV	2012	China	Wild type	94.6	98.5	98	97.3	96.6
40	MH381810.1	GTPV-AV41	GTPV	2018	China	vaccine	94.7	98.5	98.1	97.3	96.6

*U* unknow; /:no data for this cell

Overall, the similarities to the full genome, two subgroups were grouped according to the P32 gene, an envelope protein of the virus, which is homologous to the P35 protein encoded by the Vaccinia virus (Vaccinia virus VV) H3L gene. The gene is located on the envelope surface of the mature virus particle. According to the nucleotide sequence of the P32 gene of LSDV, the homology of the LSDV/NMG/2020 strain with the SPPV strains is 98%, the homology with the GTPV strains is 98-98.3%, and the homology with the LSDV strain is 99.1 − 100%. The nucleotide sequence homology between LSDV/NMG/2020 and LSDV vaccine strains of South Africa was 99.8%. The homology with Russia vaccine-like recombinant LSDV/Russia/Udmurtiya/2019 (MT134042.1) strain is 99.1%. The homology with the LSDV/Xinjiang/2019 (MN598006.1) strain is 100%. LSDV/NMG/2020 strain and all other strains isolated in China along with the 20L43Ly-Quoc/VITM/20(MZ577074.1) strain belonged to the same subgenotype by full-genome analysis.

The RPO30 gene is a nucleotide sequence homologous gene of the vaccinia virus E4L gene, which encodes a DNA-dependent RNA polymerase subunit and plays a role in virus replication [24]. Comparisons between the LSDV strains generated in China with the LSDV vaccine strains obtained in South Africa, the homology of the RPO30 gene sequence was 99.7%. By analysis of the EEV gene of 27 bp insertions/deletion, all strains obtained in China were grouped into the same group. With reference to the nucleotide sequence of the LSDV EEV gene, the homology of the LSDV/NMG/2020 strain and the SPPV strain is 97.1%, the GTPV strain is 97.1–97.3% and the LSDV strain is 94.1-100%. The homology of the nucleotide sequence is 99.8% between LSDV/NMG/2020 and LSDV vaccine strains from South Africa. The homology with the Russia vaccine-like recombinant virus LSDV/Russia/Udmurtiya/2019 (MT134042.1) is 99.8%. The homology with the China/GD01/2020 strain (MW355944.1) is 100%.

Capripoxvirus G-protein-coupled chemokine receptor (GPCR) was supposed as a suitable gene for capripoxvirus discrimination as the GPCR of field type strains has a deletion of about 12 bp compared with the vaccine strain [25]. The LSDV/NMG/2020 strain has a 12-bp insertion similar to the vaccine strain. In this study, the field strains were clustered into distinct lineages by GPCR analysis. Our results suggested that the highest homology of the LSDV strains isolated in China was China/GD01/2020 (MW355944.1), LSDV/Hongkong/2020 (MW732649.1), and 20L43_Ly-Quoc/VNM/20 (MZ577074.1). We analyzed the LSDV117 gene, which was rarely reported previously, according to the nucleotide sequence of the LSDV117 gene of LSDV, the LSDV/NMG/2020 shares significant homology with Russia vaccine-like recombinant virus LSDV/Russia/Udmurtiya/2019 (MT134042.1) is 99.8%, and different lineage with the Neethling strain and LSDV/Saratov/2017.

We conclude that the LSDV/NMG/2020 isolate belongs to a new emerging subgenotype of the LSDV in the genus Capripoxvirus. Further study to evaluate the biological and epidemiological features of the strain can establish effective control programs for LSD outbreaks.

## Data Availability

The data that support the findings of this study are available from the corresponding author upon reasonable request. The datasets generated and/or analysed during the current study are available in the NCBI GenBank database repository, [OL977078 (GPCR), OL977075 (EEV), OL977074 (P32), OL977077 (LSDV117), OL977076 (RPO30), DN616408 (Lumpy skin disease virus strain LSDV/NMG/2020, complete genome)].
